# Species-Specific Codon Context Rules Unveil Non-Neutrality Effects of Synonymous Mutations

**DOI:** 10.1371/journal.pone.0026817

**Published:** 2011-10-26

**Authors:** Gabriela R. Moura, Miguel Pinheiro, Adelaide Freitas, José L. Oliveira, Jörg C. Frommlet, Laura Carreto, Ana R. Soares, Ana R. Bezerra, Manuel A. S. Santos

**Affiliations:** 1 RNA Biology Laboratory, Department of Biology and CESAM, University of Aveiro, Aveiro, Portugal; 2 Institute of Electronics and Telematics Engineering (IEETA), University of Aveiro, Aveiro, Portugal; 3 Department of Mathematics, University of Aveiro, Aveiro, Portugal; University of Umeå, Sweden

## Abstract

**Background:**

Codon pair usage (codon context) is a species specific gene primary structure feature whose evolutionary and functional roles are poorly understood. The data available show that codon-context has direct impact on both translation accuracy and efficiency, but one does not yet understand how it affects these two translation variables or whether context biases shape gene evolution.

**Methodologies/Principal Findings:**

Here we study codon-context biases using a set of 72 orthologous highly conserved genes from bacteria, archaea, fungi and high eukaryotes to identify 7 distinct groups of codon context rules. We show that synonymous mutations, i.e., neutral mutations that occur in synonymous codons of codon-pairs, are selected to maintain context biases and that non-synonymous mutations, i.e., non-neutral mutations that alter protein amino acid sequences, are also under selective pressure to preserve codon-context biases.

**Conclusions:**

Since *in vivo* studies provide evidence for a role of codon context on decoding fidelity in *E. coli* and for decoding efficiency in mammalian cells, our data support the hypothesis that, like codon usage, codon context modulates the evolution of gene primary structure and fine tunes the structure of open reading frames for high genome translational fidelity and efficiency in the 3 domains of life.

## Introduction

The degenerate nature of the genetic code introduces flexibility in gene evolution, allowing for selection of coding sequences with high stability and translational efficiency. Various studies uncovered biases in codon usage associated to translational selection in practically all living beings (reviewed in [1], [2]). Thus, single codons are not chosen randomly, they are under the influence of a number of factors that modulate both speed and accuracy of protein synthesis. The distribution of codon-pairs is also not random but it is independent of codon-usage biases [3], [4], indicating that these two gene primary structure variables evolve independently.

Codon-context biases have been studied in Eubacteria, Archaea and Eukaryota [5], [6] and both general and kingdom-specific trends which can be attributed to translational efficiency and accuracy have been discovered [7]–[11]. However, mutational pressure and epigenetic regulatory features also play a role in codon-context evolution [12]–[15]. Obviously, codon-context features associated to mRNA translation are only found in protein coding genes, while nucleotide context features linked to other selective forces, namely G+C pressure, are found in coding and non-coding regions.

In order to clarify the role of codon context in gene translation and gene evolution and to simplify de novo gene design algorithms, we have studied codon context in conserved and highly expressed genes where translational biases are stronger and more easily identifiable. Our approach is based on the following hypothesis: “if codon context modulates mRNA decoding efficiency then the features that modulate translation efficiency should be visible in highly expressed genes”. Similar approaches have been successfully used to identify functional rare codons that play roles in protein folding [16]. With this goal in mind, we have carried out multiple alignments of orthologous genes from bacteria, archaea and eukaryotic species and developed software tools to highlight codon-context features in these alignments. This allowed us to identify subsets of conserved codon contexts that shape the evolution of coding sequences. We demonstrate here that some of these conservation patterns are so strong that can divide codon contexts into well defined sub-groups. The overall study shows that codon context is a punctual modulator of coding sequence evolution and that context conservation alone is sufficient to explain changes in mRNA and polypeptide sequences. We also show that codon-context imposes positive pressure on synonymous codons and that apparently neutral mutations are in fact constrained by the need to maintain codon-context patterns.

### Results

### Codon-context bias in conserved genes

**Figure 1:**
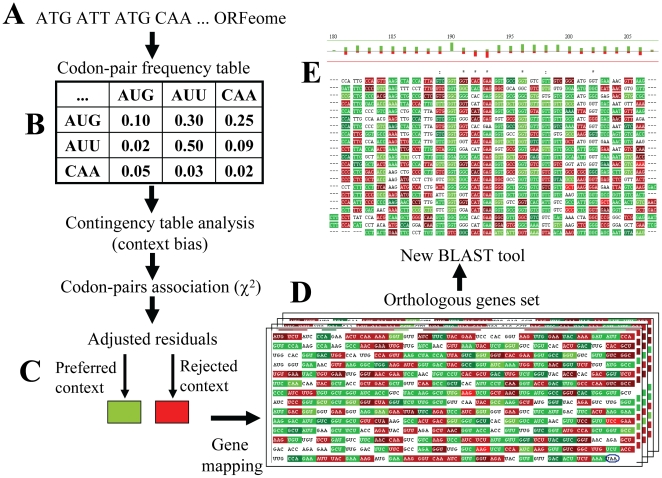
Schematic representation of the bioinformatics pipeline used to study codon context. **A)** ORFeome sequences were downloaded from public genome databases (Figure S1) and were stored using a local database. **B)** Our software package Anaconda performed an initial quality control step where ORFs were filtered, only those that started with an AUG, ended with an in frame stop codon and had no internal stops or undetermined nucleotides (N) were selected. Valid ORFs were then used in downstream analyses of each ORFeome. Codon-pair 61x64 contingency tables for each ORFeome were then built and analysed. These tables included the observed frequency of every possible pair of codons identified by the combination of lines (first codon) and columns (second codon) and were used to test the null hypothesis of non-association using Chi-square tests and adjusted residuals values. **C)** Codon-pairs responsible for that association, i.e. those with adjusted residuals above 5.00 or bellow −5.00, are highlighted using a color-code scale where green indicates preference and red indicates rejection relative to non-association. This approach was developed and validated before by our group and has been described in detail elsewhere [4], [5], [17], [38]. **D)** The color scale obtained for each organism was used to highlight codon context preferences at the gene level for the entire set of orthologous genes. **E)** Sequences annotated with codon-context biases were then aligned using a BLAST tool implemented in Anaconda that allows for identification of conserved and non-conserved codon-context patterns in the orthologous gene set alignment.

In order to determine whether codon context is conserved in coding sequences, a group of 72 highly expressed genes from *S. cerevisiae* (Table 1) was used to retrieve orthologs from the 3 domains of life (Figure S1). The codon context biases were then determined as previously described by Moura and colleagues [4], [17]. Multiple alignments of the orthologous genes allowed us to highlight codon contexts in red, black and green, according to the bias detected ([4], [17], Figure 1).

**Table 1:** Gene set.

ADH1
ASC1
CCW12
CDC19
ENO1
FBA1
GPM1
ILV5
PGK1
TDH1
TPI1
RP(L,P,S) – 61 GENES

*S. cerevisiae* genes that were used to build the orthologous gene list analyzed in this study. Individual ribosomal proteins (RP in the table) are included as supporting information (Figure S7).

**Figure 2:**
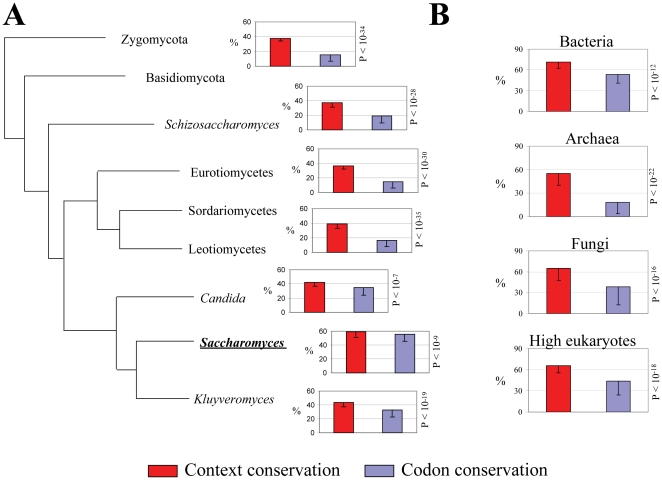
Codon-pair contexts are highly conserved. **A)** Codon-context conservation in fungi was determined by calculating the percentage of preferred or repressed codon contexts that showed bias conservation in the aligned sequences of each organism versus *S. cerevisiae* (red bars in the graphs). The percentage of conservation of the first codon of the pair between both sequences is shown in blue bars. This allows for comparison of codon-context and first codon conservation in the alignments. The graphs are organized according to the fungal phylogenetic tree described by Fitzpatrick and colleagues [39]. One fungal species was chosen for each branch of the tree, as follows: Zygomycota/Basidiomycota – *C. neoformans*; *Schizosaccharomyces* – *S. pombe*; Eurotiomycetes – *A. fumigatus*; Sordariomycetes/Leotiomycetes – *N. crassa*; *Candida* – *C. tropicalis*; *Saccharomyces* – *S. mikatae*; *Kluyveromyces* – *E. gossypii*. **B)** The same approach was used to compare codon-context and first codon conservation in a group of bacteria, archaea and high eukaryotes (see Figure S1), using *E. coli*, *M. jannaschii* and *H. sapiens* as reference organisms, respectively. In order to determine the statistical significance of the data the plots were tested using two-tailed T-student tests for paired samples, and all organisms showed significantly higher conservation of codon contexts than first codon conservation (10^−35^<p<10^−7^).

**Figure 3:**
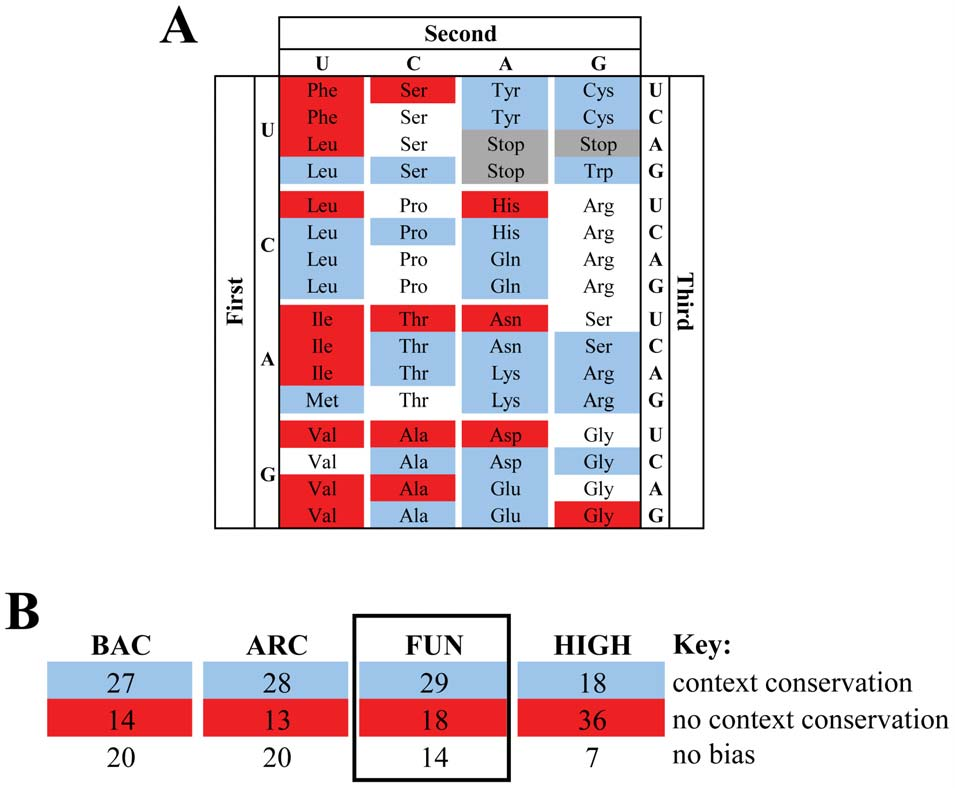
The conservation of codon context depends on the nature of the codon-pair and on phylogeny. **A)** In order to elucidate how codon-context conservation is maintained, the percentages of conserved codon contexts were calculated for each codon-pair starting with each possible codon, represented here in a genetic code table format (e.g. results for the UUU-NNN codon-pair family are shown at the top-left corner of the panel). Whenever the percentage of conserved codon pairs was higher than the percentage of non-conserved pairs (difference >4%), the codon was colored in blue, otherwise codons were colored in red. The remaining cases were considered non-biased and were kept uncolored. Using the previous example, since 24% of the UUU-NNN codon pairs exhibited codon context conservation while 76% were not conserved, the respective square was colored red in the panel. **B)** The total number of blue/red/white codon-pair types of the 4 phylogenetic groups shows that high eukaryotes have the highest number of non-conserved codon contexts (36), while the other 3 groups showed more conserved than non-conserved contexts.

Codon-pairs used in the orthologs of each group of organisms (bacteria, archaea, fungi and high eukaryotes) were then compared with those of *E. coli*, *M. jannaschii*, *S. cerevisiae* and *H. sapiens*, respectively, to determine codon context conservation (Methods and Figure 2). Determination of the conservation of single codons of codon-pairs showed a significant number of scenarios where the first codon changed but the context color was conserved (Figure 2). In order to determine if those differences were related to the nature of the two codons of codon-pairs, data from bacteria, archaea, fungi and high eukaryotes were re-analyzed by separating the codon-pairs by their first codon and searching again for context conservation. As shown in Figure 3A for fungal species and in Figure 3B and Figure S2 for the 4 phylogenetic groups, the pattern of codon context conservation among the 4 groups was rather different, with more codon-context pairs being conserved in bacteria (44%), archaea (46%) and fungi (48%) than in high eukaryotes (30%) (Figure 3B, Figure S2). Also, the number of unbiased pairs of codons decreased from bacteria (33%) to high eukaryotes (11%) (Figure 3B, Figure S2).

**Figure 4:**
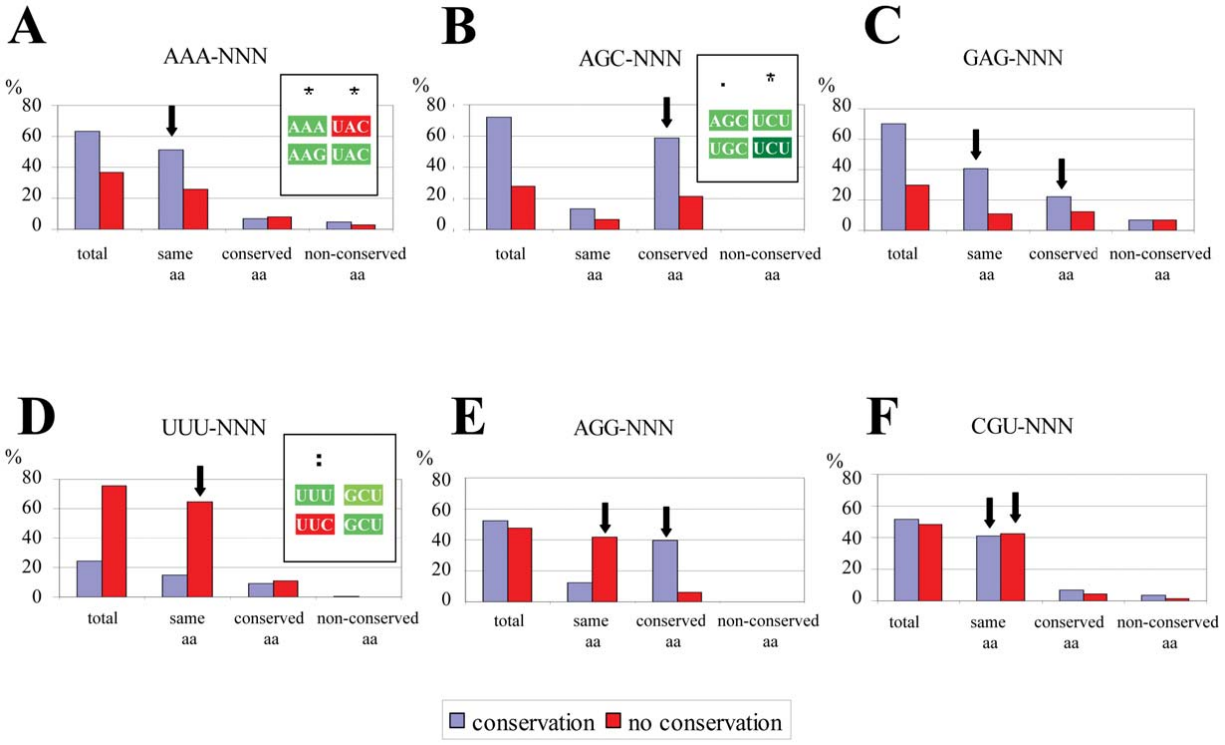
Fungal codon-context conservation is organized into specific patterns. In order to clarify why codon-context conservation is stronger than single codon conservation (first codon of a pair of codons), the multiple alignment of the orthologous genes set was reanalyzed and the conservation pattern of single codons was determined. For this, the percentage of conserved and non-conserved codon contexts for each codon pair family (represented as “total”) was divided in 3 sub-categories of codon variation, namely: i) the first codon of the pair changed to synonymous codons (“same aa”), ii) the first codon of the pair changed to a conserved amino acid (“conserved aa”) or, iii) the first codon of the pair changed to encode non-conserved amino acids (“non-conserved aa”). The analyses for all possible codon-pairs identified 7 different patterns, 6 of which are exemplified here. **A)** Context conservation was mainly maintained by changing the first codon of the pair to a synonymous codon (see example in inset, where the green context AAA-UAC of *S. cerevisiae* is aligned to the codon pair AAG-UAC, which is also green in the test fungal species); **B)** Context conservation was mainly maintained through changing an amino acid to a conserved one (example in inset, where the substitution of an AGC-Ser codon by a UGC-Cys allowed for color maintenance); **C)** Context conservation was maintained through a combination of the two previous strategies as highlighted by the arrows; **D)** The first codon changed to a synonymous codon and altered the context (example in inset, where the green UUU-GCU context of *S. cerevisiae* corresponds to a red UUC-GCU context in the test species); **E)** The first codon changed to a synonymous codon and changed the context or the first codon changed to encode a conserved amino acid and the context was maintained (arrows) ; **F)** The amino acid was maintained, independently of the context bias. The 7^th^ group is not shown in the figure and includes all codon-pairs with insufficient information or no clear conservation trend. The insets of panels A, B and D were extracted from the alignment performed by Anaconda.

**Figure 5:**
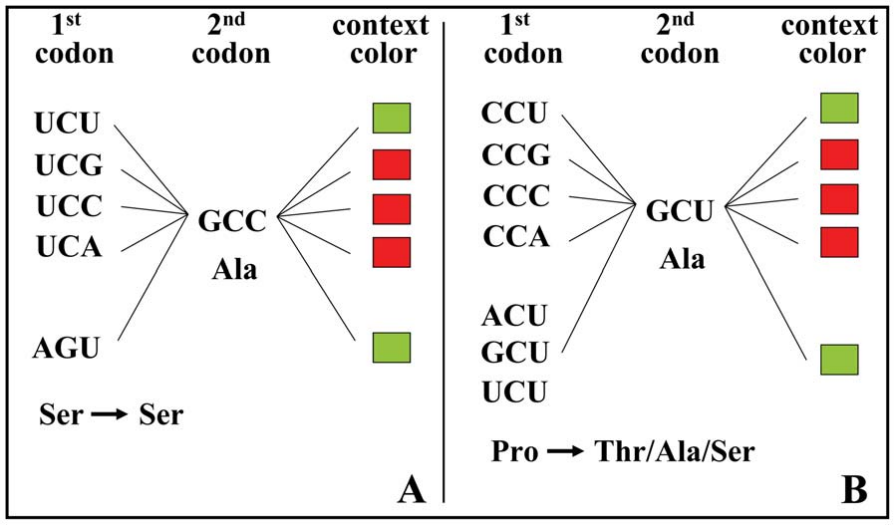
Codon context model. This model explains how the first codon of a codon-pair affects the outcome of the codon-context bias. The 3^rd^ nucleotide (N_3_) of the first codon of codon-pairs is the major modulator of the codon-context bias (adjusted residual value), as has already been shown by our group in a previous study (see text). Changing this nucleotide can revert the context signal of a particular codon pair, turning it from positive to negative or vice versa. For example, a U-ending codon such as UCU next to the Ala GCC codon produces a preferred context, conversely to the other UCN codons (panel A). In order to maintain the context signal, the first codon of the pair is sometimes altered to another codon ending with the same nucleotide of the original first codon (AGU in the example of panel A), even if the amino acid is different (ACU, GCU or UCU in the example of panel B). In cases where there is more than one synonymous codon resulting in the same context signal (e.g. AGU and UCU), first codon alteration may maintain the amino acid (panel A). The figure shows codon context biases of *S. cerevisiae* as an example to illustrate the concept outlined in the model.

In order to identify the codon mutational dynamics responsible for this codon-context conservation, the original data set was split again into 3 different groups depending on the variation of the first codon, namely: i) codons that changed to synonymous ones; ii) codons that changed to codons belonging to conserved amino acids; or iii) codons that changed to codons belonging to non-conserved amino acids. In fungi, codon-contexts were mainly conserved when the amino acids of the pair were not altered (Figure 4A,C) or when the codons changed to non-synonymous codons of conserved amino acid families (Figure 4B). Significant differences were not detected when codons changed to codons encoding chemically distinct amino acids (non-conserved amino acids). Conversely, the context was not conserved mainly when codons changed to synonymous codons in the first position of the pair (Figure 4D). This result can be explained by the fact that synonymous codons usually share the first two nucleotides and differ in the third one only. Indeed, previous studies have shown that the major codon-context preferences are associated with the X_3_-Y_1_ di-nucleotides of codon-pairs X_1_X_2_X_3_-Y_1_Y_2_Y_3_ (see [4], [5]) and, therefore, a change in X_3_ may reverse the context color (see Figure 5A). This also explains why changes from synonymous to non-synonymous codons belonging to similar amino acids may maintain the context; X_3_ nucleotides may remain unchanged (Figure 5B).

### Codon-pairs can be grouped by context patterns

**Figure 6:**
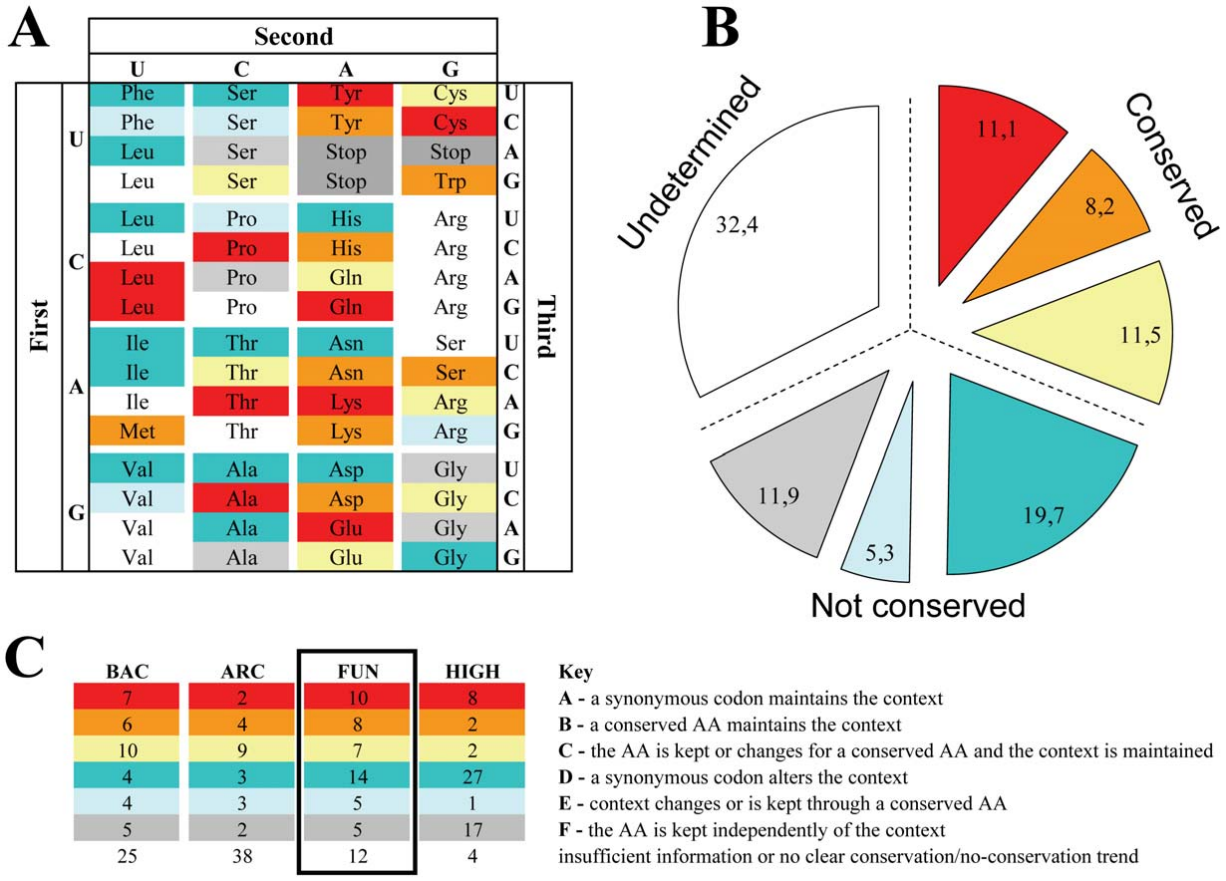
Specific patterns single codon and codon-context conservation. In order to characterize the patterns of conservation of single codons and codon-pairs in the 4 phylogenetic groups, the percentage of conserved and non-conserved codon contexts of all types of codon pairs was subdivided in 3 sub-categories of codon variation, namely: i) the first codon of the pair changed to synonymous codons, ii) the first codon of the pair changed to codons of a conserved amino acid, or iii) the first codon of the pair changed to codons of non-conserved amino acids. This approach (exemplified in Figure 4), allowed for identification of 7 context patterns which covered all codon-pairs starting with each possible codon, represented here as a genetic code table (as in Figure 3A). To each type of conservation pattern we have attributed a different color as explained in the key of panel C. **A)** Shows the context pattern distribution in fungal species. **B)** Shows the percentage of codon pairs belonging to the different color patterns among all species. **C)** Shows the relative contribution of each phylogenetic group to the total values shown in B). Archaea have higher percentage of unbiased contexts, fungal contexts are mainly conserved and high eukaryotic contexts are mainly non-conserved.

In an attempt to elucidate the maintenance of codon-context patterns in cases where neutral synonymous codons changed and in cases involving chemically similar amino acids, the codon-pairs plots of the four phylogenetic groups were arranged according to the pattern of conservation of each codon pair, i.e. each plot was compared to those shown in Figure 4 and was classified using a color code to highlight each group, as in Figure 3A. This allowed us to classify the codon-pairs in 7 major patterns, the 7^th^ group included codon-pairs that did not produce significant biases (Figure 6, Figure S3). In fungi (Figure 6A), codon-pairs starting with the codons AAA-Lys, ACA-Thr, CAG-Gln, CCC-Pro, CUA-Leu, CUG-Leu, GAA-Glu, GCC-Ala, UAU-Tyr and UGC-Cys normally altered the first codon to a synonymous codon to maintain the context (plot A in Figure 4; red squares in Figure 6). Codon pairs starting with the codons AAU-Asn, ACU-Thr, AUC-Ile, AUU-Ile, CAU-His, CUU-Leu, GAU-Asp, GCA-Ala, GCU-Ala, GGG-Gly, GUU-Val, UCU-Ser, UUA-Leu and UUU-Phe changed the first codon mainly to a synonymous codon and altered the context (plot D in Figure 4; blue squares in Figure 6). This was the case for the majority of codon contexts in high eukaryotes (Figure 6C, Figure S3) and explained the low codon-context conservation of this group of organisms. In order to further clarify the genome representability of each of these context patterns, the number of codon-pair types of each pattern (Figure 6C) was displayed in a pie chart (Figure 6B). Almost one third of the contexts were conserved (red, orange and yellow) while the other two thirds were either not conserved (blue and grey) or were undetermined (white) (Figure 6B).

The contribution of each phylogenetic group to those scores was diverse. For example, high eukaryotes contributed with the highest number of non-conserved contexts 18%, against 5%, 3% and 10% of bacteria, archaea and fungi, respectively. Fungi and bacteria contributed with the highest number of conserved contexts, 10% and 9%, respectively, while archaea showed the highest proportion of unbiased results (16%). In other words, the majority of codon contexts in high eukaryotes were non-conserved while most codon-pairs in bacteria and fungi were conserved and most codon-pairs in archaea showed no bias. The latter was probably related to small sample size and poorer quality of the alignments due to lower similarity of orthologues. This is supported by the fact that archaeal genes showed the highest frequency of complete context changes, i.e. the number of times both codons of the codon-pairs changed (Figure S4).

### Codon-context biases modulate evolution of coding sequences

**Figure 7:**
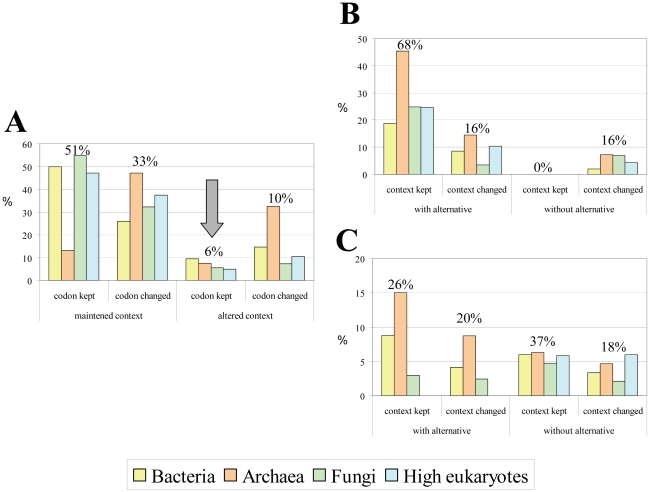
Coding sequences evolve to maintain codon-context patterns. In order to determine whether evolution of gene sequences is influenced by codon-pair conservation, we have compared the behavior of the first codon of codon-pairs with codon-context bias conservation in the ORFeomes whose genes were under analysis. All panels show the percentage of events in bacteria, archaea, fungi and high eukaryotes and the relative percentages calculated using data from all organisms is shown above the bars. **A)** The panel shows the percentage of cases where the first codon of a pair was conserved (“codon kept”) or not conserved (“codon changed”) in organisms that maintained the context bias, or altered it. For example, the codon pair AGU-AAU which is preferred by *S. cerevisiae* (adjusted residue  = 10), is rejected by *S. pombe* (adjusted residue  =  −20). In such cases (right part of the graph) the codon-pair was seldom maintained (6%), suggesting that there was selective pressure to recover the original context bias. **B)** The cases where the first codon of the pair changed to a synonymous codon were divided depending on whether the context was conserved (“context kept”) or not (“context changed”), and whether there was a synonymous codon that would allow for context conservation (“with alternative”) or not (“without alternative”). Whenever there was a synonymous codon that allowed for context bias conservation, that codon was used preferentially, since a significantly high portion of those cases kept the context (68%). **C)** When the first codon of the pair changed to a codon belonging to a conserved amino acid, the codon-context biases were mainly conserved (26+37 = 63%), with only 20+18 = 38% of non-conservation. The level of context bias conservation was especially high when there was no synonymous codon that would allow for amino acid conservation without changing the context bias (i.e. “without alternative” – 37%).

In order to determine the relevance of codon-context conservation for the evolution of coding sequences, the codon-pairs associated with codon-context conservation in each group of organisms were further studied (i.e. red, orange and yellow colored codon-pairs in Figure 6 and Figure S3). We investigated first whether codon-pairs that altered their context, i.e., identical codon pairs (e.g. AGA-ACC) that had different colors in the reference and test organisms, were more prone to mutate in order to recover the original color/bias (to achieve context conservation). For this, we have calculated the frequency of unchanged or changed codon pairs, depending on whether the color of the original pair was maintained or reversed, relative to the reference genome (Figure 7A). The frequency of codon conservation in cases where the codon-context was altered (arrow in Figure 7A) was significantly lower than that of the other 3 possibilities, suggesting that context created positive selective pressure on the codon-pair. We have then isolated codon-pairs where we could detect alteration of the first codon of the pair to a synonymous codon to determine whether synonymous codons appeared randomly or whether positive mutational pressure selected codons that maintained the context bias. Whenever a synonymous alternative maintained the context bias it was selected (Figure 7B). Indeed, 68% of the first codon alterations maintained the color and only 16% showed color alteration (p = 5.39E-10). The percentage of synonymous codon alterations at the first codon position which resulted in alteration of context color was identical in cases where an alternative synonymous codon maintained the context (16%) and when such alternative did not exist (16%), suggesting that random codon alterations represent 16% of possible mutations only.

Codon pairs where the first codon mutated to another codon belonging to a conserved amino acid family were also analyzed in the same way (Figure 7C). This allowed us to test whether the choice of a different amino acid could be explained by the need to maintain codon context. Again, 63% (26%+37%) of the mutations involving conserved amino acids maintained the color of the codon-pair, while only in 38% (20%+18%) of the cases a color change was detected (p = 8.5E-05). Moreover, in those 63% of conserved contexts involving conserved amino acids more than half (37%) could be explained by the absence of a synonymous codon that could maintain the context pattern. Therefore, in cases of codon-pairs where synonymous codon alternatives did not exist, amino acids rather than context color were altered, a result that further supported the relevance of codon-pair context bias in the evolution of coding sequences.

## Discussion

Gene translation accuracy is a conserved feature of life [18]. Analyses of variables which are commonly used to quantify evolutionary change and gene expression variation show conserved patterns of covariation which are amenable to computational simulation. Such studies have shown that protein misfolding associated to translational misreading explains most of the codon usage biases observed in highly expressed genes [18]. Indeed, genes apparently evolve to avoid mRNA mistranslations and protein misfolding, which is mainly achieved through selection of optimally translated codons, in particular in conserved protein domains. This trend is dependent on gene dispensability (i.e. fitness effect associated to gene deletion) and on the sensitivity of cells or tissues towards protein misfolding [18].

Like codon usage, the context of codons is implicated in translational speed and accuracy, but in ways that are apparently stronger than codon usage [9]. Indeed, specific codon contexts are implicated in missense [7], [19], nonsense [20]–[23] and frameshifting errors [24]–[26] which in turn are influenced by environmental cues, such as the amino acid supply [7], [27]. Certain contexts are also repressed because of ribosome slippage during ribosome decoding [28]. Recently, the relevance of codon-pair contexts on translational efficiency has been highlighted in a study where Synthetic Attenuated Virus Engineering (SAVE) was used for production of live attenuated viral vaccines [10], [11]. In this study viral genomes were redesigned by substituting frequent with infrequently used codon-pairs (codon context) without changing the codon usage bias of the gene nor the amino acid composition of proteins. These recombinant viruses did not show major changes in their *in vitro* growth rates, but produced less protein than wild type viruses and their virulence was attenuated in mice infection models. Indeed, these codon context engineered viruses were still able to replicate inside the host, did not cause significant symptoms during infection, but were effective in mice immunization [11]. Therefore, much like codon usage, codon context modulates the efficiency of protein synthesis, although the exact molecular mechanism behind this phenomenon is still unclear. Furthermore, one does not yet understand whether context effects are restricted to specific domains of coding sequences or are felt along the entire length of mRNA. That almost 1/3 of the codon-pair types are conserved (Figure 6B) supports the hypothesis that context effects influence translation along the entire length of mRNAs. Our data shows that codon-context conservation, from bacteria to vertebrates, is mainly achieved through the preferential utilization of synonymous codons or amino acids with similar chemical properties.

Important implications of defined codon context patterns are the imposition of specific constraints on the evolution of coding sequences and non-neutrality effects of most mutations. Indeed, synonymous mutations that alter codon-context patterns are highly likely to affect translation efficiency. We have demonstrated that codon pairs that altered their context bias tend to accumulate additional mutations in order to restore sequence-specific codon context biases. Therefore, codon context and codon usage biases [29], local variation in gene expression [30] and fitness penalties associated to mistranslation [31], should all be included in the calculation of the number of synonymous nucleotide substitutions per synonymous site (dS) in order to estimate more accurately the rate of neutral evolution.

Various groups have addressed the problem of the origin of biased genome G+C content in bacterial genomes [32], [33], and have shown that strong biases favoring G+C rich mutations are counterbalanced in a second mutational step by purifying selection [34]. One possible implication of our data is that codon context could also affect G+C content of coding sequences, however, we were unable to detect specific trends that could support this hypothesis (Figure S5).

A significant number of codon contexts (2/3 of the total) were not conserved in our analysis, either because there was not enough data to allow for bias determination (1/3 of the cases), or because they were affected by specific mutational pressures or epigenetic constraints [12]–[15]. Interestingly, 100% of the NNU-NNN contexts of high eukaryotes, 63% of fungi, 25% of archaea and 19% of bacteria, were not conserved, reinforcing the discriminatory power of the U_3_N_1_ dinucleotides in codon-context biases [4]–[6].

Also interesting was the low codon-context conservation observed in high eukaryotes relative to the other phylogenetic groups (Figures S2, S3). This apparent softening of evolutionary pressure is not observed in codon usage [18] and suggests that multi-cellular organisms may use somewhat different mRNA decoding rules. It is possible that codon contexts become less important for translational accuracy in high eukaryotes because their higher number of tRNA isoacceptor genes may increase cognate codon decoding (see Figure S6 and [35]). This is supported by the observation that error-prone codon contexts are often associated with codons that are read by rare and/or near-cognate tRNAs (e.g. [36], [37]). If so, codon context can be further distinguished from codon usage, as the latter is mainly dependent on tRNA abundance rather than cognate codon-anticodon interactions.

In conclusion, almost one third of all codon-pairs from bacteria, archaea and eukarya have a significant tendency to conserve context biases in essential genes even if such conservation requires mutations that alter amino acid sequences. Therefore, codon context modulates gene primary structure evolution and, more importantly, neutral mutations that alter codon context create strong negative translational pressure on the codon pair forcing the introduction of addicional mutations that restore species-specific codon context biases.

## Methods

### Retrieval of orthologous genes

ORFeome sequences were retrieved from NCBI Genbank (ftp.ncbi.nih.gov/genomes/), the Broad Institute (www.broad.mit.edu/annotation/), the *Candida* Genome database (www.candidagenome.org), www.nature.com/nature/journal and the Ensembl ftp site (ftp.ensembl.org/pub/current_fasta/) (see Figure S1 for details and links). For retrieval of orthologuous gene sets, the sequences of 72 highly conserved *S. cerevisiae* genes (Table 1 and Figure S7) were downloaded from SGD (http://www.yeastgenome.org/) and were aligned using Anaconda (species listed in Figure S1). All best matches were then aligned against the entire ORFeome of *S. cerevisiae* and only reciprocal best matches were considered. The *K. waltii* ORFeome had a reduced number of valid ORFs, and in order to increase this number an additional BLAST was performed using *K. lactis* orthologues against *K. waltii* GenBank sequences (using the tool for genomic blast against fungi). These putative *K. waltii* orthologues were then aligned against the *K. lactis* ORFeome and only reciprocal best matches were considered. All alignments and subsequent sequence handling took into consideration the alternative nuclear genetic code of *C. albicans*, *C. tropicalis*, *D. hansenii*, *C. guilliermondii* and *C. lusitaniae* (leucine CUG codons are decoded as serine in these species). ORF sequences that did not start with an ATG codon, did not end with one of the 3 stop codons (TAA, TGA, TAG), had internal stop codons or undefined bases (N), were discarded from the dataset using Anaconda tools for sequence quality control [17].

### Conservation measurements

Each group of orthologous genes was uploaded into Anaconda and was mapped according the codon-context biases identified for each organism (Figure 1), as described previously [4], [5], [17]. Briefly, Anaconda counts all codon pairs of each complete set of coding sequences (ORFeome) and classifies them as preferred or repressed relative to what would be expected if both codons were associated independently. This statistical discrimination is shown by the adjusted residue value which is positive for preferred and negative for rejected codon pairs. This methodology allows for mapping codon-pair context biases at the ORFeome level. Figure 1D shows an example of a mapped ORF, highlighting preferred codon contexts in green and repressed contexts in red. Since adjusted residues are calculated by analysing the frequency of two consecutive codons, color overlapping was eliminated by coloring the first codon of the pair only, the second codon is the first of the next pair of codons.

The mapped ORFs were then aligned using the BLASTP multiple-alignment tool which was implemented in Anaconda [38] with the following parameters: maximum E-value  = 0.5; GOP = 11; GEP = 1; matrix  =  BLOSUM62; identity  =  more than 10%; ORF aligned  =  more than 0%. Alignments such as those shown in Figure 1E were used to count the number of times the first codon of a pair and/or the context biases changed, i.e. from red to green or vice-versa, when compared to the reference sequence. In order to simplify the analysis, all bacterial sequences were compared to those of *E. coli*, and *M. jannaschii*, *S. cerevisiae* and *H. sapiens* sequences were used as references for the archaeal, fungal and high eukaryotic genes, respectively. Changes of the first codon of a pair were considered according to the BLASTP output, i.e., codons that remained unchanged (*), codons that changed to synonymous ones (:), codons that changed to conserved amino acids (.), or codons that changed to non-conserved amino acid families ( ). Codon-context changes were considered whenever a significantly high bias (adjusted residual above 5.00) changed to a significantly low one (adjusted residual bellow −5.00), or vice versa (see [4], [17] for statistical details). We tested whether the high codon usage bias of the orthologous genes used influenced the representation of codon-pairs. For this, the number of codons that were absent in the dataset was counted. The results show an averaged effective number of codons close to 61 and a global codon coverage close to 100% (Figure S8).

### Statistics

Significant differences in single codon and codon-pair conservation between test and reference samples were determined using Microsoft Excel spreadsheets and SPSS software tools. The statistical tests used were either two-tailed T-student tests for paired samples (as in Figure 2) or ANOVA analyses for two-factors without replication, followed by post-hoc tests (as in Figure 7). Whenever the number of species per group was low (e.g. n = 5), non-parametric (Wilcoxon) tests were used in parallel with ANOVA analyses, with similar outcomes. The adjustment of proportions to the normal distribution was tested through Kolmogorov-Smirnov (KS) tests prior to further analyses. Values were considered significantly different if p<0.05, except for simultaneous T-student tests where α was divided by the total number of tests performed, according to the Bonferroni's correction to avoid the artificial increase of the α parameter.

## Supporting Information

Figure S1 **Data sources.** ORFeome sequences were downloaded from the public databases indicated in the table. Each ORFeome was scanned and checked for non-valid ORFs (see Methods) and used for orthologues retrieval and codon-context analyses using our software package Anaconda.
(TIF)

Figure S2 **Patterns of codon-context conservation in the four phylogenetic groups.** Context conservation percentages were calculated separately for each codon-pair starting with each possible codon (represented here in a genetic code table format). For example, results for the UUU-NNN codon-pair family are shown at the top-left corner of the 4 panels. Whenever the percentage of conserved pairs of a codon-pair family was higher than the percentage of non-conserved pairs (difference >4%) the position of its first codon was colored in blue in the respective panel. Whenever the non-conserved percentage exceeded the conserved one by 4% or more the corresponding codon was colored in red. The remaining cases were considered as non-biased and were kept uncolored. For comparative purposes, the analysis was carried out separately for the 4 phylogenetic groups and the panels are shown together: BAC – bacteria; ARC – archaea; FUN – fungi; HIGH – high eukaryotes. The lower panel shows the number of blue/red/white codon-pair families in each phylogenetic group.
(TIF)

Figure S3 **Specific preferences of codon context and single codon conservation.** After characterizing the conservation pattern of all possible codon-pairs in the 4 phylogenetic groups, 7 different possibilities emerged (as exemplified in Figure 4). Each type of conservation pattern corresponds to a particular color (as explained in the key at the bottom of the figure) and the codon pairs are identified by their first codon and displayed in the genetic code table format. The results obtained for the majority of codon pairs starting with UUU are shown at the top-right corner of the panels. For comparative purposes, the analysis was carried out separately for the 4 phylogenetic groups and the panels shown correspond to: BAC – bacteria; ARC – archaea; FUN – fungi; HIGH – high eukaryotes. The lower panel shows the number of codons belonging to each pattern in each phylogenetic group.
(TIF)

Figure S4 **Proportion of 4 possible outcomes of codon-pair conservation.** In order to quantify the codon coverage achieved by this study, we have computed the percentage of unchanged pairs, the percentage of pairs in which both codons changed and the two intermediate possibilities. The percentages are plotted separately for each phylogenetic group and the global percentages are indicated above each pattern: • - • – codons did not change in the aligned sequence when compared to the reference sequence; X - • – only the first codon of the pair changed; • - X – only the second codon of the pair changed; X - X – both codons changed. Most of the studies herein described focused on codon pairs where only the first codon differed between the two aligned sequences (arrow).
(TIF)

Figure S5 **Codon context does not influence genome G+C content.** In order to elucidate if codon context conservation contributed somehow to genome G+C content the nucleotide variation identified in the orthologous gene alignments were divided in 3 groups, depending on whether they enriched G+C content, A+T content or maintained G+C/A+T. Data was also grouped according to the effect of mutations on codon context bias (maintained, altered or both) and also according to the mutated position in the codon pair. For example, 1_4 corresponds to mutations that affect either X_1_ or Y_1_ of the hexanucleotide X_1_X_2_X_3_-Y_1_Y_2_Y_3_. An average value is presented above the bars for each group of results. The pattern is identical in cases where the context is maintained, when it changes or when both situations are plotted together, meaning that codon context conservation does not influence G+C content. Globally, the percentage of A+T enrichment is always higher than that of G+C enrichment (with an average difference of 9%), as a consequence of a higher G+C content of reference ORFeomes when compared to most test species (data not shown). As expected, positions 3_6 are the most prone to change while positions 2_5 are the most conserved ones.
(TIF)

Figure S6 **The number of codons decoded by cognate anticodons increases from bacteria to vertebrates.** In order to quantify cognate and non-cognate codon decoding, the number of codons with cognate anticodons was determined (data extracted from Genomic tRNA Database at http://lowelab.ucsc.edu/GtRNAdb/), averages and standard deviations were calculated for each phylogenetic group and compared using single factor ANOVA followed by post-hoc tests. Cognate decoding was significantly lower in bacteria and higher in high eukaryotes than in the other 3 groups (P<0.05). The total number of tRNA genes for each species was also averaged and is shown inside the bars.
(TIF)

Figure S7 **List of** ***S. cerevisiae*** **ribosomal protein genes.** The sequences of the 61 ribosomal proteins shown here were used to build the orthologous gene sets for this study.
(TIF)

Figure S8 **Codon coverage of the analyses.** In order to evaluate whether the high codon usage bias of the orthologous gene list used distorted the representation of codon-pairs of in these sequences were counted for all species. The results show that a very small number of codons were absent in the dataset (4^th^ column), yielding an averaged effective number of codons close to 61 and a global codon coverage close to 100%. The most limited dataset was the *K. waltii* for which only 20 ORFs were retrieved, but still more than 87% of the codons were present (highlighted in red).
(TIF)
