# Correction: Species-Specific Codon Context Rules Unveil Non-Neutrality Effects of Synonymous Mutations

**DOI:** 10.1371/journal.pone.0145593

**Published:** 2015-12-17

**Authors:** Gabriela R. Moura, Miguel Pinheiro, Adelaide Freitas, José L. Oliveira, Jörg C. Frommlet, Laura Carreto, Ana R. Soares, Ana R. Bezerra, Manuel A. S. Santos

There are errors in the Funding section. The correct funding information is as follows: This study was partially funded by the EU-FP7 MEPHITIS project, by FEDER funds through COMPETE (FCOMP-01-0124-FEDER-014284) and by National Funds through FCT—Foundation for Science and Technology in the scope of the research projects PTDC/BIA-BCM/72251/2006, PTDC/BIA-BCM/64745/2006 and PTDC/BIA-GEN/110383/2009. The Institute of Electronics and Telematics Engineering of Aveiro (IEETA) supported the development of the Anaconda software package. The funders had no role in study design, data collection and analysis, decision to publish, or preparation of the manuscript.
