# Supplementary figures and images for: Species-Specific Codon Context Rules Unveil Non-Neutrality Effects of Synonymous Mutations
Source: PLoS One. 2011 Oct 26;6(10):e26817. doi: 10.1371/journal.pone.0026817 (PMC3202573; doi:10.1371/journal.pone.0026817)

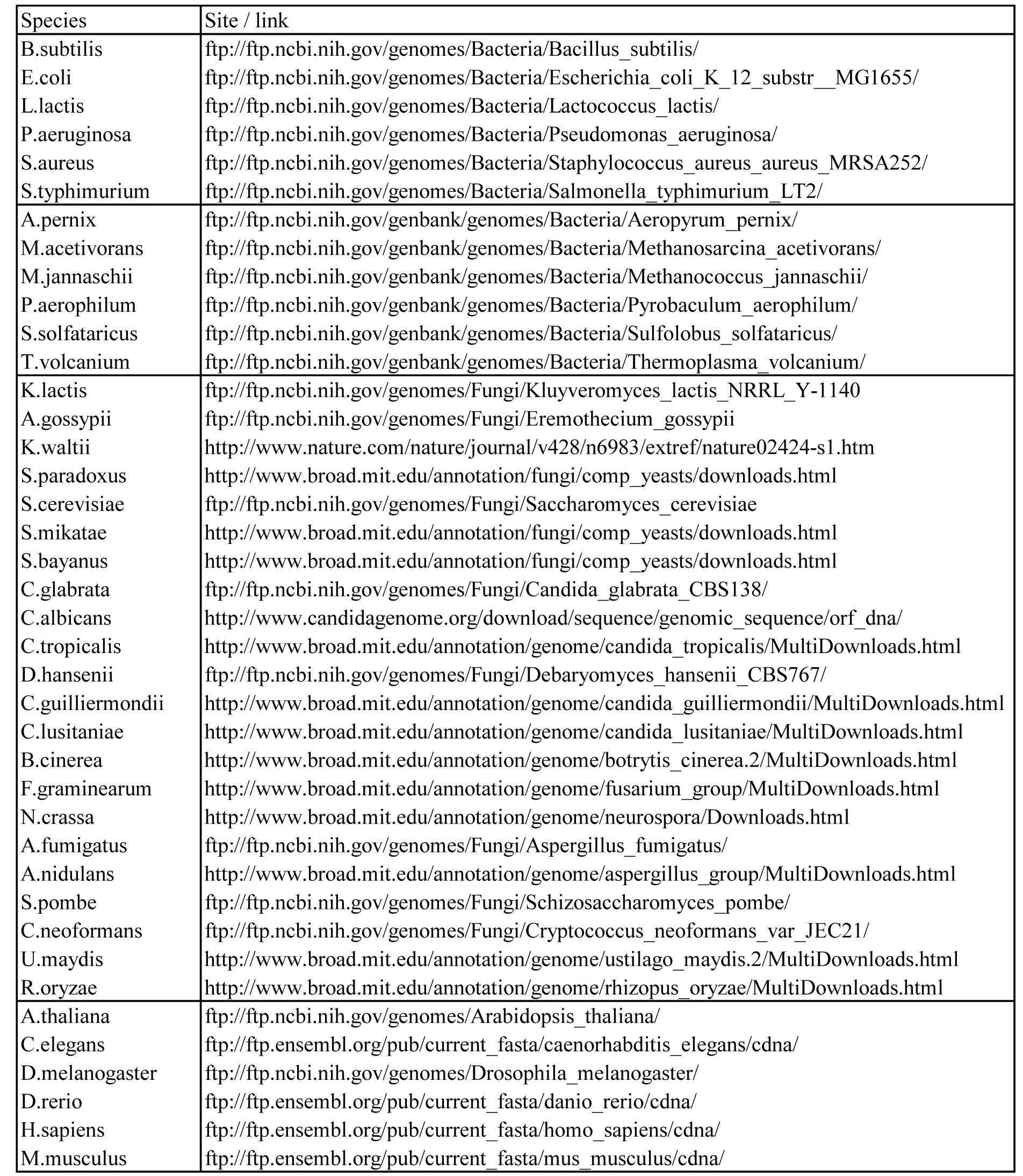

Supplement: Figure S1 — Data sources. ORFeome sequences were downloaded from the public databases indicated in the table. Each ORFeome was scanned and checked for non-valid ORFs (see Methods) and used for orthologues retrieval and codon-context analyses using our software package Anaconda. (TIF) [file pone.0026817.s001.tif]

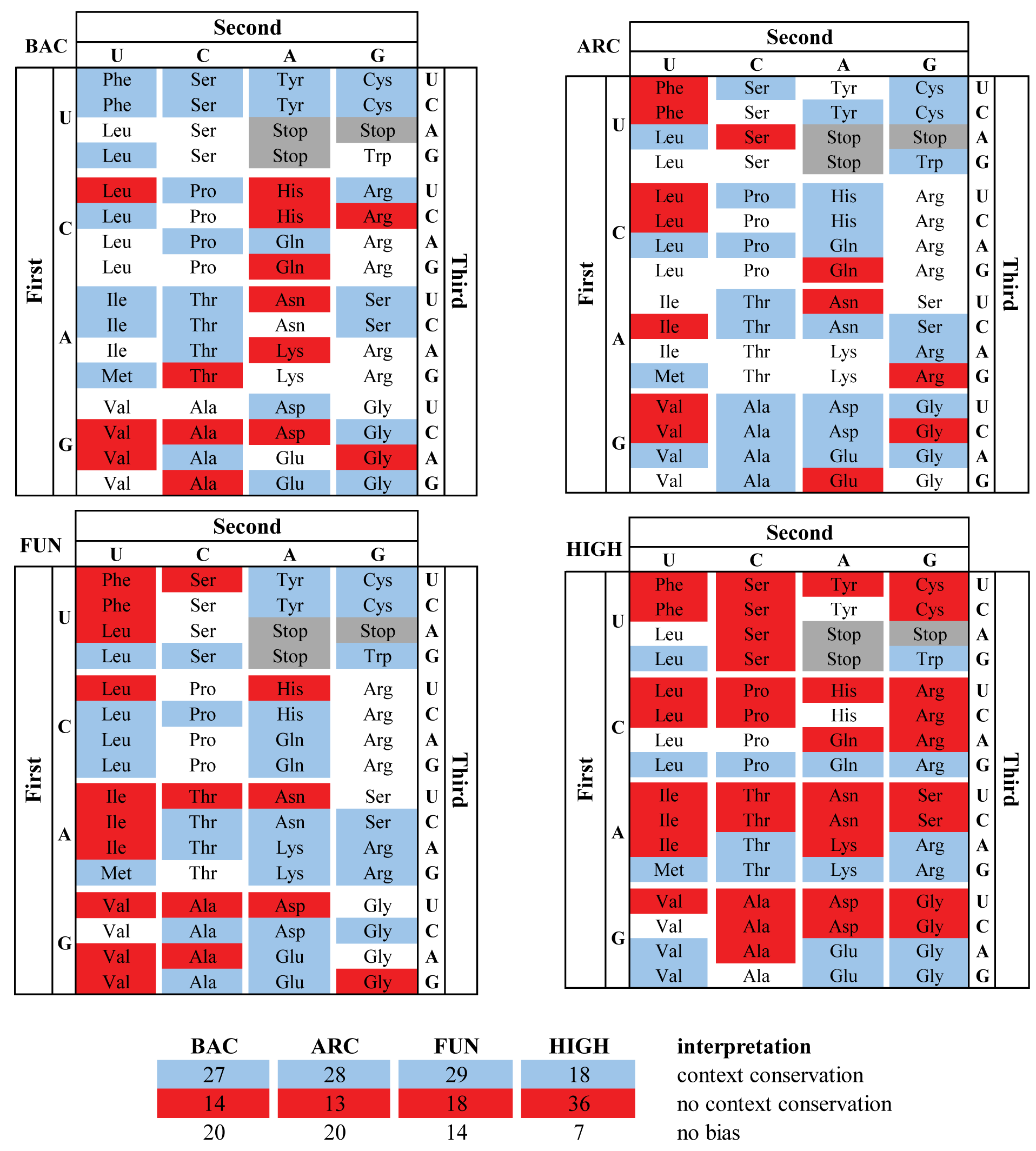

Supplement: Figure S2 — Patterns of codon-context conservation in the four phylogenetic groups. Context conservation percentages were calculated separately for each codon-pair starting with each possible codon (represented here in a genetic code table format). For example, results for the UUU-NNN codon-pair family are shown at the top-left corner of the 4 panels. Whenever the percentage of conserved pairs of a codon-pair family was higher than the percentage of non-conserved pairs (difference >4%) the position of its first codon was colored in blue in the respective panel. Whenever the non-conserved percentage exceeded the conserved one by 4% or more the corresponding codon was colored in red. The remaining cases were considered as non-biased and were kept uncolored. For comparative purposes, the analysis was carried out separately for the 4 phylogenetic groups and the panels are shown together: BAC – bacteria; ARC – archaea; FUN – fungi; HIGH – high eukaryotes. The lower panel shows the number of blue/red/white codon-pair families in each phylogenetic group. (TIF) [file pone.0026817.s002.tif]

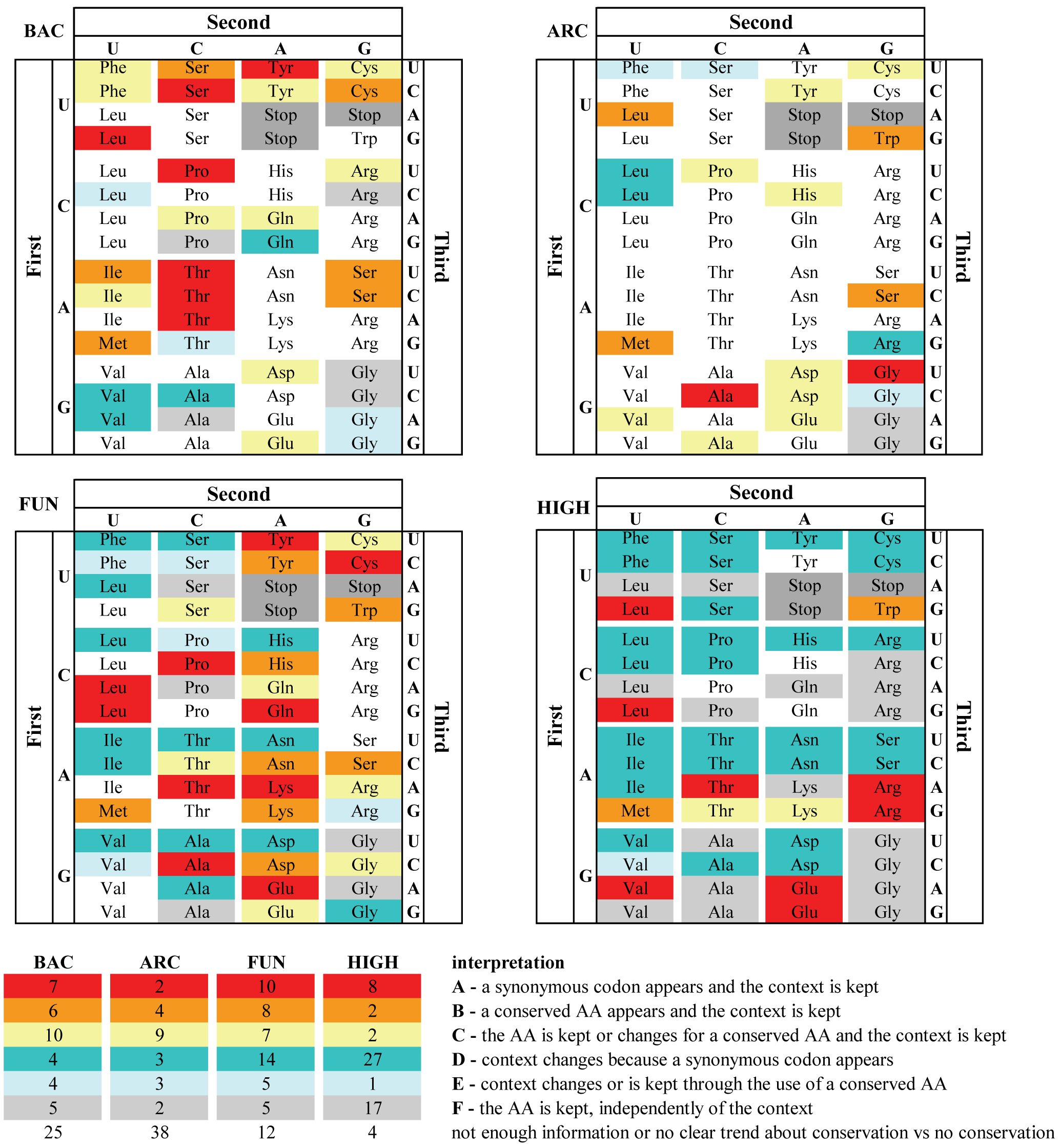

Supplement: Figure S3 — Specific preferences of codon context and single codon conservation. After characterizing the conservation pattern of all possible codon-pairs in the 4 phylogenetic groups, 7 different possibilities emerged (as exemplified in Figure 4). Each type of conservation pattern corresponds to a particular color (as explained in the key at the bottom of the figure) and the codon pairs are identified by their first codon and displayed in the genetic code table format. The results obtained for the majority of codon pairs starting with UUU are shown at the top-right corner of the panels. For comparative purposes, the analysis was carried out separately for the 4 phylogenetic groups and the panels shown correspond to: BAC – bacteria; ARC – archaea; FUN – fungi; HIGH – high eukaryotes. The lower panel shows the number of codons belonging to each pattern in each phylogenetic group. (TIF) [file pone.0026817.s003.tif]

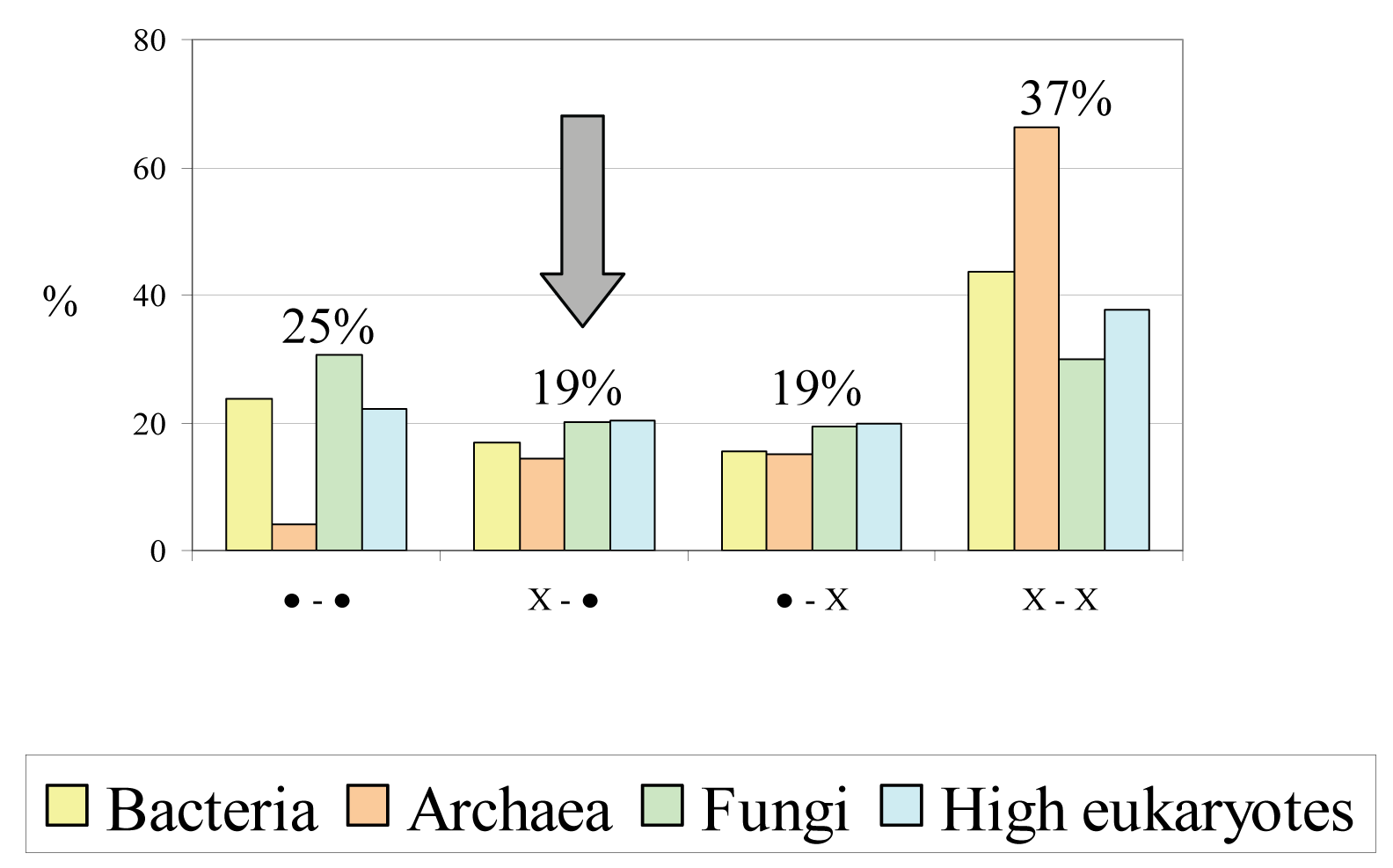

Supplement: Figure S4 — Proportion of 4 possible outcomes of codon-pair conservation. In order to quantify the codon coverage achieved by this study, we have computed the percentage of unchanged pairs, the percentage of pairs in which both codons changed and the two intermediate possibilities. The percentages are plotted separately for each phylogenetic group and the global percentages are indicated above each pattern: • - • – codons did not change in the aligned sequence when compared to the reference sequence; X - • – only the first codon of the pair changed; • - X – only the second codon of the pair changed; X - X – both codons changed. Most of the studies herein described focused on codon pairs where only the first codon differed between the two aligned sequences (arrow). (TIF) [file pone.0026817.s004.tif]

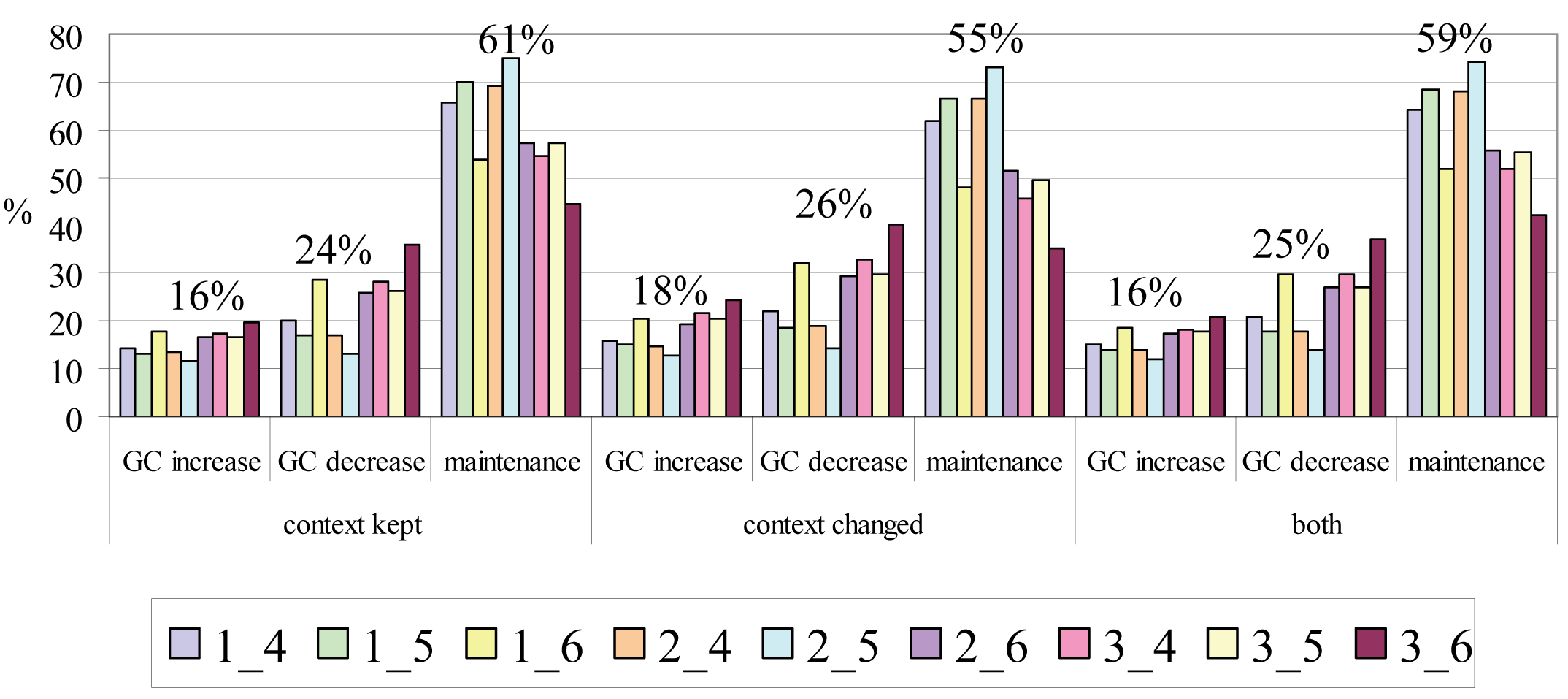

Supplement: Figure S5 — Codon context does not influence genome G+C content. In order to elucidate if codon context conservation contributed somehow to genome G+C content the nucleotide variation identified in the orthologous gene alignments were divided in 3 groups, depending on whether they enriched G+C content, A+T content or maintained G+C/A+T. Data was also grouped according to the effect of mutations on codon context bias (maintained, altered or both) and also according to the mutated position in the codon pair. For example, 1_4 corresponds to mutations that affect either X1 or Y1 of the hexanucleotide X1X2X3-Y1Y2Y3. An average value is presented above the bars for each group of results. The pattern is identical in cases where the context is maintained, when it changes or when both situations are plotted together, meaning that codon context conservation does not influence G+C content. Globally, the percentage of A+T enrichment is always higher than that of G+C enrichment (with an average difference of 9%), as a consequence of a higher G+C content of reference ORFeomes when compared to most test species (data not shown). As expected, positions 3_6 are the most prone to change while positions 2_5 are the most conserved ones. (TIF) [file pone.0026817.s005.tif]

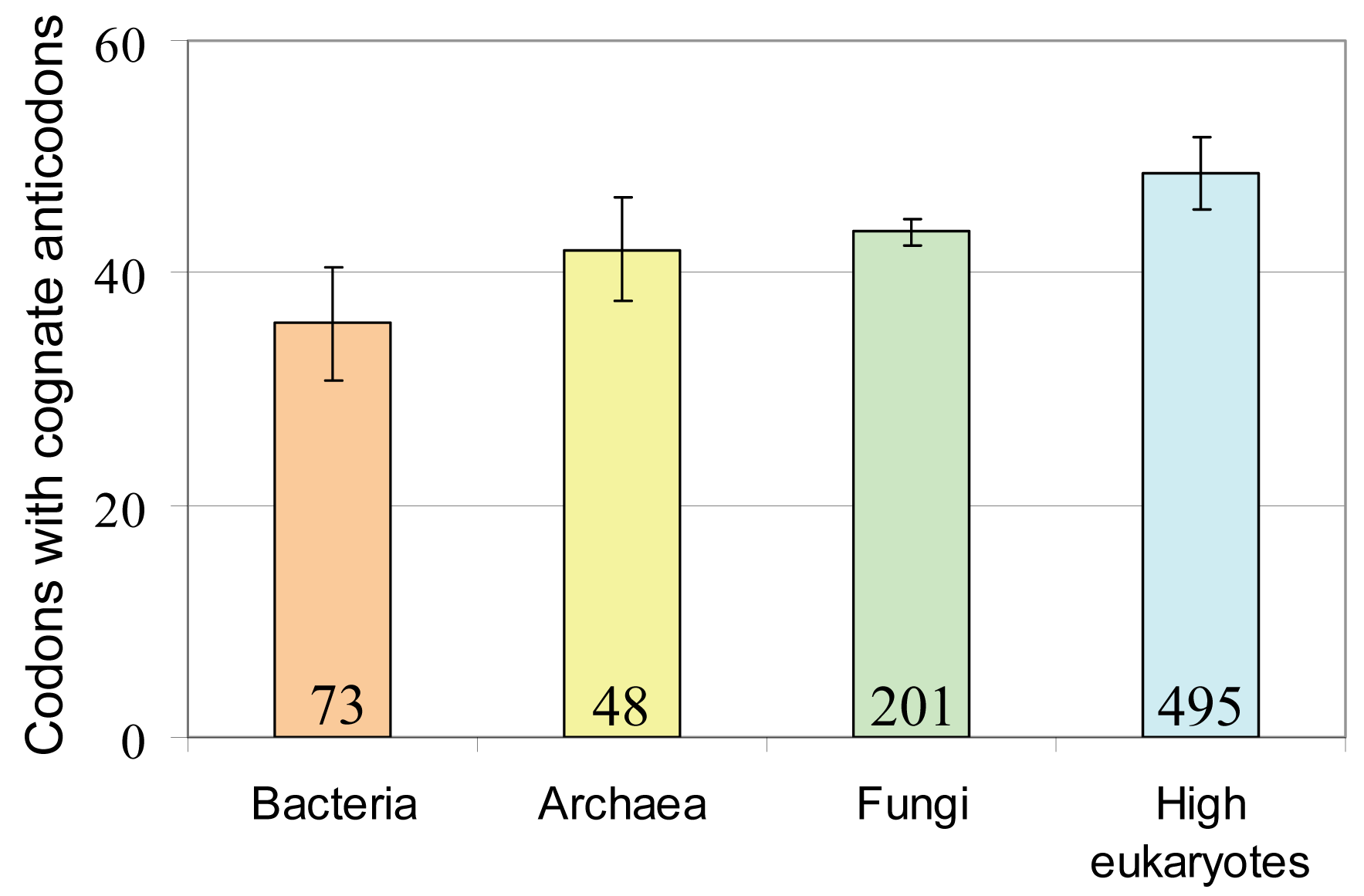

Supplement: Figure S6 — The number of codons decoded by cognate anticodons increases from bacteria to vertebrates. In order to quantify cognate and non-cognate codon decoding, the number of codons with cognate anticodons was determined (data extracted from Genomic tRNA Database at http://lowelab.ucsc.edu/GtRNAdb/), averages and standard deviations were calculated for each phylogenetic group and compared using single factor ANOVA followed by post-hoc tests. Cognate decoding was significantly lower in bacteria and higher in high eukaryotes than in the other 3 groups (P<0.05). The total number of tRNA genes for each species was also averaged and is shown inside the bars. (TIF) [file pone.0026817.s006.tif]

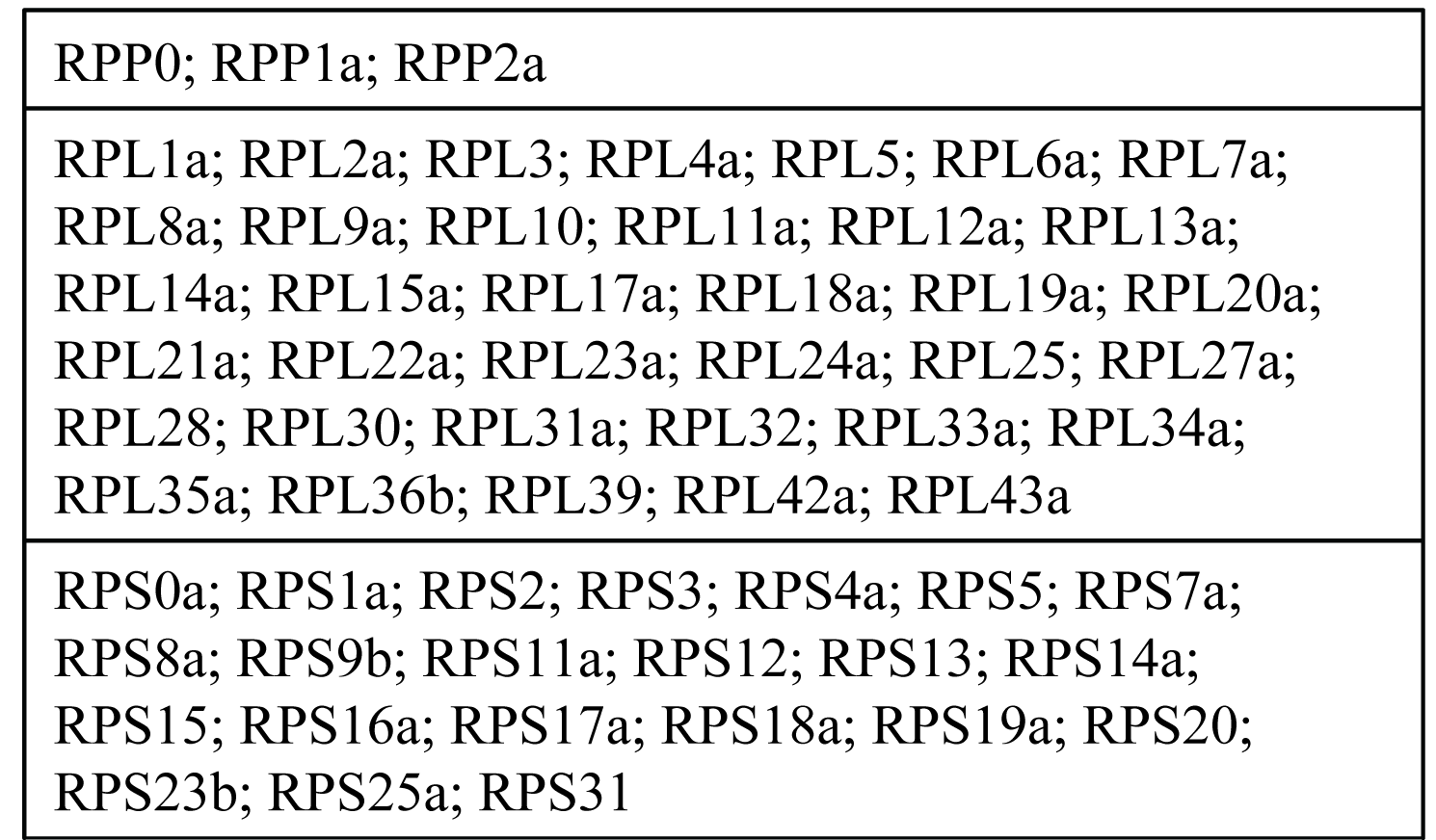

Supplement: Figure S7 — List of S. cerevisiae ribosomal protein genes. The sequences of the 61 ribosomal proteins shown here were used to build the orthologous gene sets for this study. (TIF) [file pone.0026817.s007.tif]

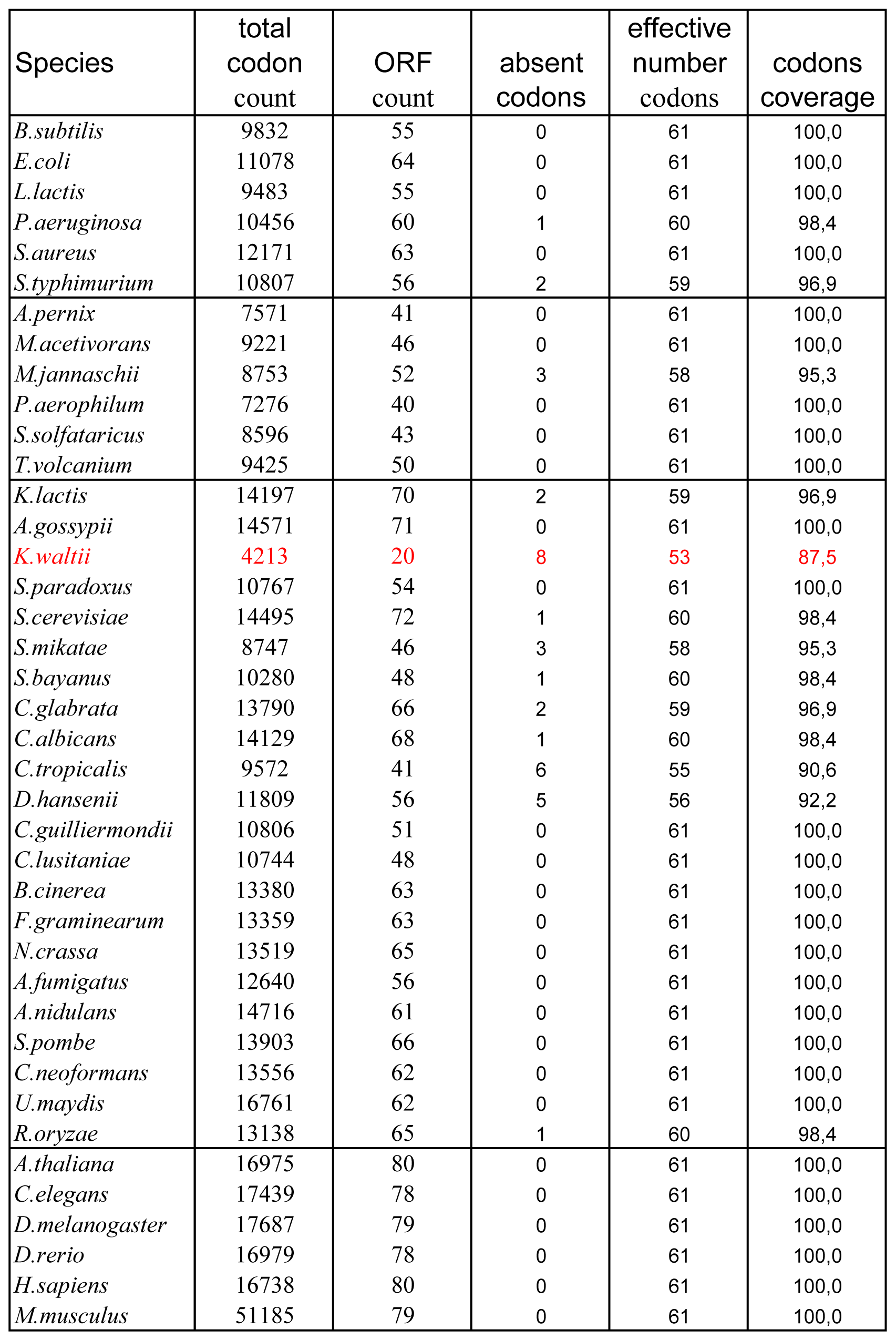

Supplement: Figure S8 — Codon coverage of the analyses. In order to evaluate whether the high codon usage bias of the orthologous gene list used distorted the representation of codon-pairs of in these sequences were counted for all species. The results show that a very small number of codons were absent in the dataset (4th column), yielding an averaged effective number of codons close to 61 and a global codon coverage close to 100%. The most limited dataset was the K. waltii for which only 20 ORFs were retrieved, but still more than 87% of the codons were present (highlighted in red). (TIF) [file pone.0026817.s008.tif]
